# Knowledge, attitude, and practices towards anthrax among Communities in the Southern and South-western Ethiopia

**DOI:** 10.1371/journal.pntd.0014848

**Published:** 2026-10-30

**Authors:** Abebe Olani, Domenico Galante, Matios Lakew, Tigist Ashagre, Taferi Benti, Gizat Almaw, Fasil Aklilu, Getnet Abie Mekonnen, Tesfaye Rufael, Nigatu Kebede, Aklilu Feleke Haile, Tadesse Eguale

**Affiliations:** 1 Department of Microbiology laboratory, Animal Health Institute, Sebeta, Ethiopia; 2 College of Veterinary Medicine and Agriculture, Addis Ababa University, Bishoftu, Ethiopia; 3 Istituto Zooprofilattico Sperimentale della Puglia e della Basilicata, Anthrax Reference Institute of Italy, Istituto Zooprofilattico Sperimentale della Puglia e Basilicata, Foggia, Italy; 4 Aklilu Lemma Institute of Health Research, Addis Ababa University, Addis Ababa, Ethiopia; 5 Ohio State Global One Health, Addis Ababa, Ethiopia; Colorado State University, UNITED STATES OF AMERICA

## Abstract

**Background:**

Anthrax caused by *Bacillus anthracis* is an endemic zoonotic disease in Ethiopia, primarily affecting domestic and wild ruminants and often resulting in severe and potentially fatal infection in humans.

**Methodology:**

A cross-sectional study was conducted from February to November 2025 to assess the knowledge, attitudes, and practices (KAP) related to anthrax among communities in 10 districts of the southern and southwestern part of Ethiopia. A total of 382 household heads aged 18 years and above were interviewed using structured questionnaires with open and closed-ended questions. Descriptive analysis, one-way analysis of variance (ANOVA), and Pearson’s correlation analysis were used to report frequenciesy, percentages and mean scores to quantify and assess associations of the knowledge, attitudes and practices of the communities toward anthrax.

**Results:**

Respondents had a mean age of 39.8 ± 12.7 years, with males comprising 92.7% of participants, and 28.3% had no formal education, while the majority were farmers (79.8%). Among the 382 participants, 60.2% had adequate knowledge. 93.7% had heard about anthrax, primarily from veterinary professionals. Two hundred twenty-seven (59.4%) of respondents were aware of the modes of transmission, 58.9% and 77.0% understood prevention measures in humans and animals, respectively. Two hundred fourteen (56.2%) of the respondents had a positive attitude towards anthrax, and 91.6% perceived anthrax as a serious disease affecting both humans and animals. A total of 187 (49.0%) of the respondents had good practices toward anthrax prevention, and vaccination practices were commonly reported, with 81.4% of them believing that vaccinating animals is important to protect human health and 98.2% to protect livestock. Among the respondents, 42.2% reported that they manage carcasses of animals suspected of sudden death by burying and burning, 36.7% reported to local veterinary authorities, 10.7% shared and ate the meat with the community and their family, and 3.6% disposed of the carcasses into the environment. Overall, the mean scores (5.9 ± 2.6 out of 10, 5.4 ± 1.2 out of 6, and 2.34 ± 1.3 out of 5) were recorded for knowledge, attitude, and practices, respectively. Participants’ knowledge mean score about anthrax was significantly associated with gender (male), level of education (degree), and occupation (government employed) (p < 0.001). There was also a positive significant correlation between knowledge-attitude (r = 0.690, p < 0.001), knowledge-practice (r = 0.417, p < 0.001), and attitude-practice (r = 0.378, p < 0.001).

**Conclusions:**

Findings from the current study provided essential insights into community awareness and preventive practices, which are critical for strengthening anthrax control and prevention strategies.

## Author summary

Anthrax is a zoonotic disease that causes illness in humans, animals, and domestic livestock, and it is endemic in Ethiopia. Livestock handling practices, including contact with sick animals, slaughtering animals, and disposing of dead animals, are major risk factors for human anthrax infection. Due to a lack of knowledge about the risks associated with improper removal of infected carcasses, the disease is not properly monitored, and socio-economic conditions contribute to underreporting and underdiagnosis of anthrax in Ethiopia. In this study, the majority of the respondents had heard about anthrax, had adequate knowledge, and held a positive attitude toward anthrax prevention within their communities. More than half of the respondents practiced good prevention measures, such as vaccinating and managing carcasses of animals suspected of dying from anthrax by burying and burning them. However, a few respondents exhibited poor practices by disposing of carcasses in the environment and sharing or consuming infected meat with their community and family. The aim of this study is to identify gaps in knowledge, attitudes, and practices (KAP) about anthrax among livestock handlers in the southern and southwestern regions of Ethiopia.

## Introduction

Anthrax caused by *Bacillus anthracis* is an endemic zoonotic disease in Ethiopia, primarily affecting domestic and wild ruminants and often resulting in severe and potentially fatal infection in humans. It is often fatal to humans [1]. It is enzootic in most countries in Africa and Asia, as well as in some countries in Europe and America [2]. Anthrax toxin and the capsule are the primary virulence factors that determine the pathogenic potential of *B. anthracis* and distinguish this pathogen from other closely related organisms [3]. The ability to form endospores, which are extremely resistant to adverse environmental factors and ability to remain viable for a long time is a key feature of *B. anthracis*, which largely determines its epidemiological potential and population structure [3].

Infection of human with anthrax occurs through the consumption of meat from infected animals and occupational exposure to infected animals or their contaminated animal products, such as wool, hides, hair, or bones [4]. Although anthrax is consistently present in certain areas, people who work with livestock in those regions often have a limited understanding of the disease [5,6]. Livestock handling practices including contact with sick animals, slaughtering animals, and disposing of dead animals, are major risk factors for human anthrax infection [7]. According to Mebratu *et al*.[8], despite livestock handlers in Ethiopia being aware of the risk of anthrax transmission to humans, 12.5% would still eat and sell the meat from an animal with suspected anthrax cases due to poor economic conditions.

Knowledge, Attitude, and Practices (KAP) survey identify knowledge, attitudes and behaviours contributing to health risks and guide the development of targeted interventions to promote healthier practices [9]. KAP surveys also reveal misconceptions and cultural influences on health behaviours. This data enables researchers to design effective strategies for promoting positive behaviour change and measuring the effectiveness of health education efforts over time [10,11].

In Ethiopia, the prevention and control of anthrax is constrained by several factors including a poor understanding of the disease dynamics, weak infrastructure, and high risk sociocultural practices. Previously published reports showed that, an outbreak of anthrax in humans due to contact with infected animals or eating infected meat of contaminated animals [12–14]. Due to a lack of knowledge about the risks associated with improper disposal of infected animal carcasses, which leads to persistent environmental contamination and recurrent outbreaks, the disease is not properly monitored, and the socio-economic conditions contribute to underreporting and under diagnosis of anthrax in Ethiopia. The main objective of this study is to identify gaps in knowledge, attitudes, and practices (KAP) about anthrax among livestock handlers in the southern, and southwestern regions of Ethiopia.

## Materials and methods

### Ethics statement

Ethical approval was obtained from the Institutional Review Board of the former Aklilu Lemma Institute of Pathobiology (ALIP IRERC 136/2016/24). Oral consent was obtained from all respondents before data collection was carried out and the data provided was not disclosed to others. We implemented stringent measures to protect participant confidentiality and ensure respondents’ privacy.

### Study area

A community-based questionnaire survey was conducted in areas of southern and southwestern Ethiopia where anthrax outbreaks have previously been reported and recurrent focal outbreaks have been documented (Fig 1). The study covered 10 districts: Benatsemay, Hammer, Bako-Dawula and Jinka in the South Ethiopia Regional State; South-Bench, Yeki, Anderacha and Masha in the Southwest Ethiopia Regional State; and Bure and Dega in the Oromia Regional State. Study areas were identified using information from active, retrospective and passive surveillance and published reports [12,15–17]. The geographic coordinates of the study area were 5.198200°–8.633825° N and 35.072540°–36.682213° E.

**Fig 1:**
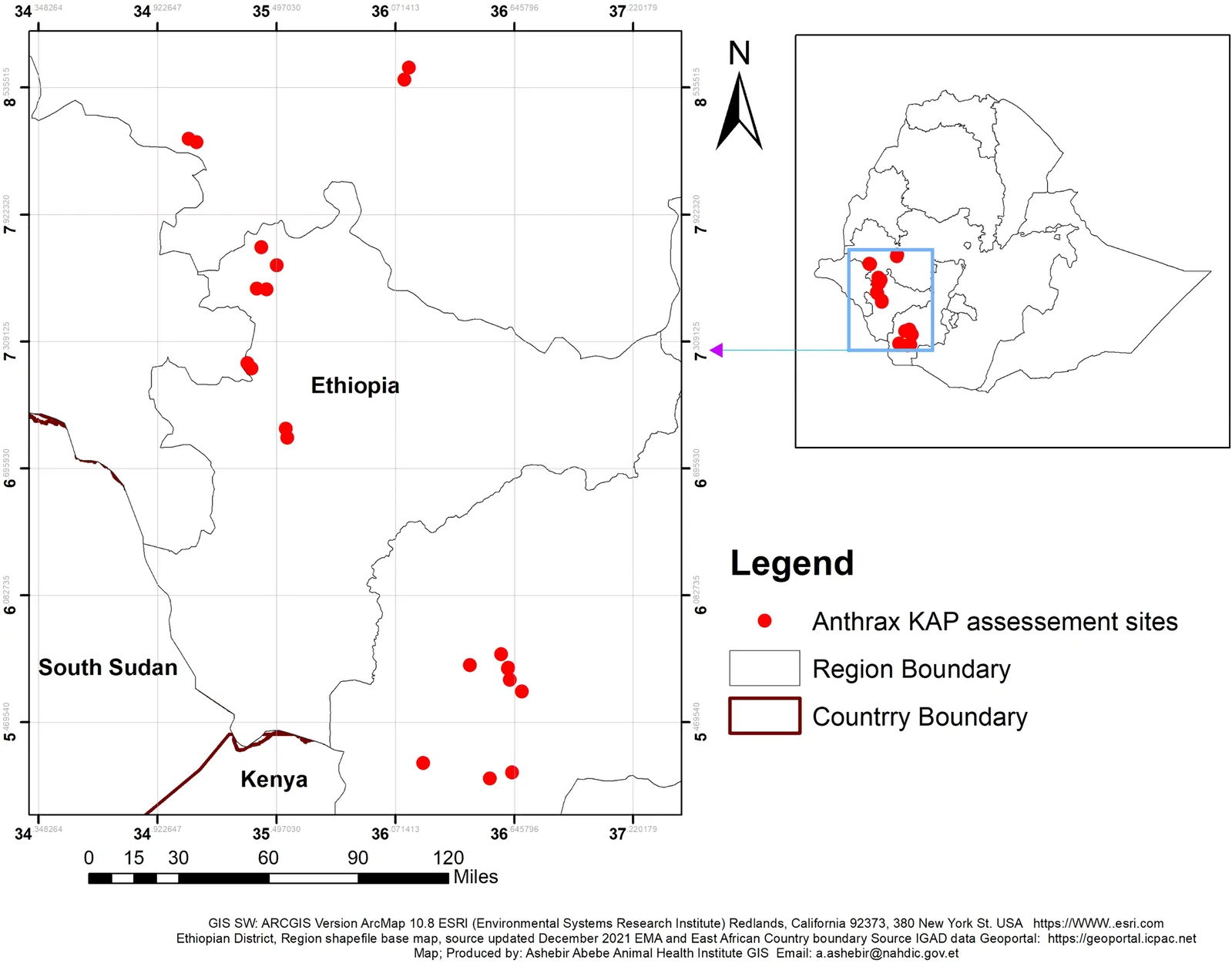
Map showing the study sites. (GIS SW: ARCGIS Version ARCMap 10.8 ESRI (Environmental System Research Institute) Redlands, California 92373, 380 New York St. USA https://WWW.esri.com; Ethiopian District, Region Shapefile base map, source updated December 2021 EMA and East Africa Country boundary Source IGAD data Geoportal: https://geoportal.icpac.net Map; produced by: Ashebir Abebe Animal Health Institute GIS Email: a.ashebir@nahdic.gov.et).

### Study population

Eligible participants were residents aged 18 years or older in the selected study areas. The study population included animal owners and community members from such as farmers, government employee, merchandising and religious leadership.

### Study design and sampling procedures

A community-based cross-sectional survey was conducted from February 2025 to November 2025 through community face-to-face interviews. A non-probability, purposive sampling strategy was used to recruit participants from areas with a history of anthrax outbreaks [18]. Districts and kebeles were treated as successive sampling units. Two kebeles were purposively selected in each of the 10 districts, taking into account the history of anthrax outbreaks and logistical feasibility, resulting in 20 kebeles. Participants were approached in these selected communities and invited to take part if they met the eligibility criteria and were willing to participate. The sampling strategy was intended to obtain information from communities with relevant experience of anthrax. Face-to-face interviewing enabled the questionnaire to be administered to participants with different literacy levels. Nevertheless, interviewer effects and social-desirability bias may have influenced responses and are considered when interpreting the findings.

### Sample size calculation and enrolment of the study participants

The sample size for this questionnaire survey was calculated using the formula given by Thrusfield [19]: with a 95% confidence level (Z = 1.96), a 5% margin of error and an assumed proportion (p) of 0.62 based on a previous report that 62% of community respondents in northern Ethiopia were aware of anthrax, a 10% non-response rate. Awareness in that study was used as a proxy for the expected proportion in the calculation; it should not be interpreted as the proportion with adequate knowledge, because having heard of anthrax is not equivalent to correctly understanding its cause, transmission, clinical manifestations or prevention [18]. A total of 382 study participants was calculated for the questionnaire survey.

### Data collection tool and procedure

Data were collected using KoboCollect (version 2025.2.3) and a structured questionnaire containing open- and closed-ended items (S1 Table). The questionnaire was developed by reviewing a previously published study and related literatures [18]. The questionnaire was translated into Amharic and Afaan Oromo and a translator was also used for participants who did not speak either language. It was translated into Amharic and Afaan Oromo; a translator assisted participants who did not speak either language. Before the main survey, the questionnaire was pretested in a rural community in Sebeta and modified to improve clarity and interpretation. The reported pretest supports an assessment of comprehensibility, but the study records provided do not describe a formal evaluation of reliability, internal consistency or construct validity..

The questionnaire covered demographic characteristics and KAP domains: 10 knowledge items addressing awareness, the cause and transmission of anthrax, signs and symptoms in humans and animals, and prevention; attitude items concerning perceived seriousness and vaccination; and five practice items concerning animal slaughter, carcass disposal and vaccination. To ensure quality, data were crosschecked independently by researchers. Face-to-face interviews were used to administer the questions. Researchers independently cross-checked collected data for quality. Potential interviewer and social-desirability effects were considered when interpreting self-reported answers.

### Scoring methods of knowledge, attitude and practices

For scoring, responses were coded as “1” for a correct answer and “0” for an incorrect or “Don’t know” answer, and the scores for each variable were summed up to provide a knowledge, attitude, and practice score for the respondents. A total mean score for knowledge, attitude, and practice was categorized based on Kabbudula *et al*. [20]. In our study, we modified the categorization for the knowledge mean score as adequate knowledge (a cut-off level of ≥6 out of 10), and good practices (≥3 out of 5) for a mean score of 60% or higher and poor for a mean score of less than 60%. Attitude was categorized as positive attitude (a cut-off level ≥ 4.5 of 6) for a mean score of 75% or higher and negative attitude for a mean score of less than 75% [21] after calculating for all respondents’ responses. Skip patterns were considered during the scoring of the KAP domains. For some questions, participants were instructed to proceed to the next step question only if they answered ‘’yes” to the preceding question.

### Data analysis

Data were downloaded from the Kobo Toolbox (https://eu.kobotoolbox.org) and imported into Microsoft Excel 2013. The dataset was then cleaned by redefining variables, removing erroneous values, and imputing missing values in preparation for descriptive and inferential statistical analysis. For data analysis, Excel data were exported to SPSS version 20.0 (IBM, Armonk, NY, USA).

Descriptive statistics were used to summarise participant characteristics and responses, including frequencies, percentages and KAP scores. One-way analysis of variance (ANOVA) was used to examine differences in mean KAP scores across demographic groups, and Pearson’s correlation coefficient was used to assess pairwise relationships among KAP scores [22,23]. Statistical significance was defined as p < 0.05. The analysis was conducted in SPSS version 20.0 (IBM, Armonk, NY, USA), following data export from Microsoft Excel. The manuscript states that missing values were imputed during data cleaning; the authors should specify which variables were affected, the imputation method and whether any sensitivity analysis was conducted.

## Results

### Socio-demographic characteristics

In this study, a total of **382 respondents** were assessed for their knowledge, attitude, and practices toward anthrax in 10 districts of southern and southwestern Ethiopia (Table 1). The mean **age of respondents was 39.8** ± **12.7 years**, and the majority were **male (91%).** Hundred eight (**27.8%)** had **no formal education**, and the highest proportion of respondents were **farmers (78.4%).** Most (91.6%) of the respondents own different domestic animals used for food sources, traction power, income and transportation purposes.

**Table 1:** Socio-demographic characteristics of the respondents (n = 382).

Characteristics	Category	Frequency(n)	Percent (%)
District	Benatsemay	50	13.1
	South-Bench	43	11.3
	Yeki	41	10.7
	Masha	40	10.5
	Bure	40	10.5
	Dega	40	10.5
	Anderacha	39	10.2
	Jinka	37	9.7
	Bako-Dawula	30	7.9
	Hamer	22	5.8
Gender(n=382)	Male	354	92.7
	Female	28	7.3
Age (years) Mean+SD* (n=382)		39.8±12.7	
Level of education (n=382)	No formal education	108	28.3
	Junior: 5-8th grade	90	23.6
	Secondary school: 9-12th grade	77	20.2
	Primary: 1-4th grade	59	15.5
	Diploma	39	10.2
	Degree	9	2.4
Occupation(n=382)	Farmer	305	79.8
	Government employee	37	9.7
	Church leaders	16	4.2
	Merchandiser	14	3.7
	Student	7	1.8
	Daily worker	3	0.8
Religion (n=382)	Christian	326	85.3
	Traditional beliefs	38	10.0
	Muslim	17	4.5
Have domestic animals (n=382)	Yes	350	91.6
	No	32	8.4

_*SD: Standard Deviation_

### Knowledge about anthrax

Findings on the assessment of knowledge regarding anthrax among the participants are shown in Table 2. Among the study population, 93.7% (n = 358) had heard about anthrax disease, and 57.3% (n = 219) primarily heard from veterinary officials. Only 13.9% (n = 53) of respondents correctly identified germs as the causative agent of anthrax, while the majority (84.8%) reported “Don’t know,” and a few (1.3%) reported “God given.” Most participants (62.8%) understood that anthrax can be transmitted from animals to humans, with 24.4% (n = 93) and 12.8% (n = 49) of the respondents reporting “Don’t know” and “No” responses, respectively. Additionally, 59.2% (n = 226) of respondents were aware of the mode of transmission, with over half of them (56.54%) having knowledge about its transmission through eating infected meat.

**Table 2:** Knowledge of community members in southern southwestern Ethiopia towards anthrax (n = 382).

Characteristics	Category	Frequency	Percent %
Ever heard about anthrax?	Yes	358	93.7
	No	24	6.3
What do you think about the cause of the disease?	Don’t know	324	84.8
	Germs	53	13.9
	God given	5	1.3
Who can be infected by anthrax?	Human and animal	223	58.4
	Animal	123	32.2
	Human	4	1
	Don’t know	28	7.3
	Wildlife	4	1
Is anthrax transmitted from animals to humans?	Yes	240	62.8
	Don’t know	93	24.3
	No	49	12.8
How do human get infected by anthrax?	Eating infected meat	216	56.5
	Slaughtering infected animals	50	13.1
	Handling infected animal products	43	11.3
	Touching infected animal faeces	38	9.9
	Drinking infected milk	35	9.2
Have you ever seen or known a person who was reported or confirmed to have anthrax?	No	291	76.2
	Yes	91	23.8
A preventive measures in animals from getting infected by anthrax	Vaccinate animals	265	69.4
	Isolate/separate anthrax infected animals	101	26.4
	Bury and burn all animal carcasses suspected of anthrax	96	25.1
	Prevent contact between infected and non-infected animals	76	19.9
	Burn all animal carcasses suspected of anthrax	62	16.2
	Bury all animal carcasses suspected of anthrax	59	15.4
	Using Traditional medicine	27	7.1
A preventive measures for human getting infected by anthrax	Avoid contact with anthrax infected animals	171	44.8
	By avoiding eating anthrax infected or suspected animal products	126	33.0
	Bury and burn all animal carcasses suspected of anthrax	103	27.0
	Vaccinate animals	76	19.9
	Bury all animal carcasses of suspected anthrax	63	16.5
	Burn all animal carcasses of suspected anthrax	46	12.0
	Using traditional medicine	18	4.7
Clinical signs /symptoms of an animal infected with anthrax	Bleeding from natural orifices	262	68.6
	Sudden death	178	46.6
	Bloating	88	23.0
	Unclotted dark red blood	55	14.4
	Other (Splenomegaly if opened)	12	3.1
	Incomplete rigor mortis	4	1.0
Clinical signs /symptoms of a person with anthrax	Skin rash/wounds	92	24.1
	Fever	56	14.7
	Fatigue (extreme tiredness)	24	6.3
	Headache	24	6.3
	Chills	16	4.2
	Diarrhoea	15	3.9
	Lack of appetite	12	3.1
	Vomiting	10	2.6
	Excessive sweating	6	1.6
	Irritability	6	1.6
	Coughing	2	0.5

In this study, 77% (n = 294) of the respondents knew about how anthrax can be prevented in animals, commonly reported as animal vaccination and burying or burning carcasses from all anthrax suspected cases. Furthermore, 58.9% (n = 225) of the respondents recognized prevention methods in humans. The majority of participants (79.8%) were aware of the clinical signs and symptoms observed in animals that die from anthrax, and 68.6% (n = 262) knew about bleeding from natural orifices, while 46.6% of the community members reported “sudden death.” However, only 28% (n = 107) of respondents knew the clinical signs and symptoms of anthrax infection in humans, with skin rash/wounds being the most common symptoms reported (24.1%). In this study, 60.2% (n = 230) of the respondents among the community in southern and southwestern Ethiopia had adequate knowledge towards anthrax, and an overall mean score of 5.9 ± 2.6 was reported (Table 2).

### Attitude of the community towards anthrax

As shown in Table 3, 60.7% (n = 232) and 91.6% (n = 350) of respondents believed that anthrax is a serious disease affecting both humans and animals, respectively. Meanwhile, 27.5% (n = 105) of the respondents believe that it does not cause serious diseases in humans, and 11.8% (n = 45) reported that they don't know whether anthrax causes serious diseases in humans or not. The majority of participants (97.9%) reported that vaccinating their animals is important to protect both animal and human health. A few (24.1%) of the respondents reported that the main reason for not always taking their animals for vaccination was the distance from the vaccination centre. Additionally, 14.9% (n = 57) stated that they were not informed, 39.9% (n = 15) cited no veterinary service, 1.8% (n = 7) of respondents reported causes of swelling following vaccination during the farming time, 1.1% (n = 4) reported causes of abortion in pregnant animals, and 0.5% (n = 2) reported that vaccination causes death of animals during drought time (Table 3).

**Table 3:** Respondent attitude towards anthrax in southern, southwestern Ethiopia, (n = 382).

Characteristic	Category	Frequency	Percentage %
Do you think that anthrax is a serious disease in humans in your locality?	Yes	232	60.7
	No	105	27.5
	Don’t know	45	11.8
Do you think that anthrax is a serious disease in animals in your locality	Yes	348	91.1
	No	10	2.6
	Don’t know	24	6.3
What prompts you to take your animals for vaccination?	To protect animals	327	85.6
	To protect humans	46	12.0
	Because others do so	9	2.4
If you do not always take your animals for vaccination, what are the reasons?	Don’t get informed when it occurs	92	24.1
	The vaccination centre is far	57	14.9
	No Veterinary services	15	3.9
	Financial problems	5	1.3
	Other	213	55.8
	Causes death during drought season	2	0.5
	Swelling of the neck during farming time	7	1.8
	Cause abortion during pregnancy	4	1.0
	No, we vaccinated our animals	200	52.4
Do you think that vaccination of animals can help to prevent anthrax in humans	Yes	311	81.4
	No	71	18.6
Do you think that vaccination of animals can help to prevent anthrax in animals?	Yes	375	98.2
	No	7	1.8

### Practise towards anthrax

Following the assessment of practices toward anthrax, 50.8% (n = 194) of the respondents practiced free grazing, 29.8% (n = 114) practiced mixed (free and zero grazing), and 22.3% (n = 89) reported that they practice zero grazing. In this study, 54.5% of the respondents had good practices when animals die suddenly. Most (42.2%) of the respondents reported managing carcasses by burying and burning them, 36.7% (n = 140) reported them to local veterinary authorities, 10.7% (n = 41) shared and ate the meat with the community and their family, and 3.6% (n = 14) of the respondents reported disposing of the carcasses into the environment. In this study, 7.3% (n = 28) of the respondents reported that either their family member or they themselves were infected with anthrax while skinning, caring for hides, and consuming meat from infected animals. Most respondents (56.0%) indicated that they vaccinate their animals against anthrax, while 39.0% (n = 149) of the respondents reported not vaccinating their animals due to a lack of information during vaccination and the long distance from the vaccination centre. Additionally, 53.7% (n = 205) reported vaccinating their animals once per year, and 46.3% (n = 177) of respondents don't know regarding the schedule of vaccination (Table 4).

**Table 4:** Assessment of practices related to anthrax among communities.

Characteristic	Category	Frequency	Percentage %
What animal husbandry do you practice? (n = 382)	Free grazing	194	50.8
	Mixed (Free and zero grazing)	114	29.8
	Zero grazing	79	20.7
	No response	10	2.6
What actions did you take when your animal die suddenly?(n = 382)	Buried /burned carcass	161	42.1
	Reported to local vet authorities	140	36.6
	Shared meat among community members	21	5.5
	Ate meat within family	20	5.2
	Disposed on the environment	14	3.7
	Other (Specify)	11	2.9
Has any member of your family ever been infected with anthrax? (n = 382)	No	323	84.6
	Don’t know	31	8.1
	Yes	28	7.3
If yes for question No 33, how did the person contract (n = 323)	Skinning dead animal	19	5.0
	Consuming infected meat	15	3.9
	Carrying hide from dead animal	9	2.4
	while carrying meat from dead animal	5	1.3
Has anthrax outbreak occurred in your area? (n = 382)	Yes	244	63.9
	No	70	18.3
	Don’t know	68	17.8
Did your animals get vaccinated get against anthrax? (n = 382)	Yes	287	75.1
	No	95	24.9
If yes, how often is animal vaccination against anthrax done in your area? (n = 287)	Once a year	205	71.4
	Do not know	82	21.5

### Association of various demographic factors with knowledge, attitude and practices among communities towards anthrax

In this study, an overall mean score of 5.9 ± 2.6 for knowledge was reported on a possible maximum of 10 questions regarding anthrax. The demographic characteristics of gender, level of education, and occupation factors had a statistically significant (p < 0.001) association with knowledge towards anthrax. Among the knowledge items listed, the highest mean score was 93.7%, “Ever heard about anthrax,” and the lowest mean score item was 13.9%, “What do you think about the cause of anthrax disease? A mean score of 5.4 ± 1.2 for attitude from 7 questions and 2.4 ± 1.3 for practice from 5 questions was reported regarding anthrax among communities in southern and southwestern, Ethiopia. There is significant mean score difference (p < 0.001) between independent variables with attitudes, and there is only a significant mean difference (p < 0.001) between level of education and practice towards anthrax (Table 5).

**Table 5:** Distribution of knowledge, attitude and practice mean scores among respondents toward anthrax in one-way ANOVA.

Demographic	Categories	Knowledge		Attitude		Practice	
		Mean±SD* score	P-value	Mean±SD^*^ score	P-value	Mean±SD^*^ score	P-value
Age (years)	19-32	5.80 ± 2.58	0.692	5.33 ± 1.2	0.796	2.28 ± 1.21	0.831
	33-47	6.11 ± 2.65		5.39 ± 1.2		2.41 ± 1.26	
	48-62	5.77 ± 2.67		5.50 ± 1.28		2.39 ± 1.25	
	63-77	5.65 ± 2.39		5.43 ± 1.16		2.30 ± 1.36	
Gender:	Female	4.18 ± 3.13	p < 0.001	4.68 ± 1.66	p < 0.000	1.93 ± 1.59	0.061
	Male	6.04 ± 2.52		5.45 ± 1.15		2.39 ± 1.21	
Level of education	Diploma	7.28 ± 2.00	p < 0.001	5.45 ± 1.15	p < 0.001	2.56 ± 0.91	p < 0.001
	Degree	8.11 ± 1.76		5.69 ± 0.80		3.00 ± 0.71	
	Junior: 5–8	5.81 ± 2.70		6.33 ± 0.71		2.31 ± 1.22	
	No formal education	5.07 ± 2.64		5.23 ± 1.25		1.91 ± 1.31	
	Primary: 1–4	5.39 ± 2.83		5.26 ± 1.31		2.37 ± 1.23	
	Secondary school: 9–12	6.60 ± 2.03		5.29 ± 1.43		2.83 ± 1.19	
Occupation	Merchandiser	6.43 ± 2.47	p < 0.001	5.50 ± 1.02	0.105	2.71 ± 1.27	0.227
	Church leader	6.38 ± 2.00		5.50 ± 0.73		2.63 ± 1.02	
	Daily worker	4.67 ± 2.08		4.67 ± 1.15		1.67 ± 0.58	
	Farmer	5.61 ± 2.66		5.33 ± 1.27		2.28 ± 1.29	
	Government employed	7.89 ± 1.58		5.92 ± 0.95		2.68 ± 0.97	
	Student	6.29 ± 2.14		5.43 ± 0.53		2.71 ± 0.95	
	Total	5.90 ± 2.61		_5.40 ± 1.21_		2.35 ± 1.25	

_*SD: Standard deviation_

Pearson’s correlation analysis findings showed (Table 6), a positive significant correlation between knowledge-attitude (r = 0.690, p < 0.001), knowledge-practice (r = 0.417, p < 0.001), and attitude-practice (r = 0.378, p < 0.001)

**Table 6:** Correlation analysis of Knowledge, attitude, and practice mean score.

	Knowledge mean score	Attitude mean score	Practice mean score
Knowledge	1		
Attitude	0.690 (p < 0.001)	1	
Practice	0.417 (p < 0.001)	0.378(p < 0.001)	1

## Discussion

This study describes anthrax-related knowledge, attitudes and reported practices among 382 adult participants recruited from 10 districts in southern and southwestern Ethiopia. The results indicate that awareness of anthrax was widespread among the surveyed participants, but knowledge was uneven across specific topics. The distinction between having heard of anthrax and understanding its aetiology is important: although 93.7% reported having heard of the disease, only 13.9% identified germs as its cause, while most respondents selected ‘don’t know’. This pattern suggests that general familiarity with the disease does not necessarily imply a sound understanding of its biological cause or the information needed to recognise and prevent infection.

In the present study, male were predominant participants (91.0%), with farmers accounting for 78.4% of the study participants. Additionally, 27.8% of respondents (n = 108) had no formal education. These sociodemographic characteristics are important when interpreting the findings on knowledge, attitudes, and practices (KAP) regarding anthrax, as sex, occupation, and educational attainment may influence access to health information, exposure to livestock-related risks, and the adoption of preventive measures.

In this study, 93.7% (n = 358) of the respondents had heard about anthrax disease, primarily from veterinary professional. Previous similar findings (94%) were reported in Kazo, Uganda [24], and in Kenya [25] reported higher (99%) rate of respondents knowing about anthrax in Kenya. In contrast to these findings, only 81% [20] and 88% reported that they heard about anthrax [26] in Zambia. The majority of respondents did not know the causative agent of anthrax, with only 13.9% (n = 53) knowing that anthrax is caused by germs, and the majority of the respondents (84.8%) reported that they don’t know. In agreement with this findings, Romha & Girmay [18] reported 77.6% in northern Ethiopia, and 72.1% in selected game management areas in Zambia [20].

Most participants involved in the current study, understood that anthrax can infect humans, domestic animals, and wildlife, and similar findings were reported in northern Ethiopia [18,27] in Zambia. Additionally, 59.4%(n = 227) of respondents were aware of the mode of transmission in which is in line with the findings from a previous study conducted in Zambia [20]. In this study, an overall mean score of 5.9 ± 2.6 for knowledge toward anthrax was reported. According to Mondal and Yamage [28], adequate knowledge is essential for early recognition, detection, and notification of the disease.

In this survey, 91.6% of respondents believed that anthrax is a serious disease affecting both humans and animals. In agreement with these findings, Kabbudula *et al*. [20]. and Senyange *et al*. [24] reported that majority of the respondents considered anthrax to be a serious disease in both animals and humans. The majority of participants reported vaccinating their animals to prevent anthrax, with 81.4% doing so to protect human health and 98.2% to protect their animals. A similar previous study in Zambia [26] reported that 82.3% of the respondents were vaccinating their animals against anthrax. In contrast to these findings, Senyange *et al.* [24] reported that 64% of respondents did not vaccinate their animals in Kazo, Uganda. Majority of the respondents vaccinate their animals to prevent anthrax but a few respondents had a negative attitude towards vaccination due to the assumption of swelling of the neck during farming time (1.8%), and abortion in pregnant animals (1.1%). Similar findings was reported [29] by previous study in the Goulburn-Murray region of Victoria, Australia.

**Relatively high levels of knowledge did not necessarily translate into consistent vaccination practices due to several possible reasons** such as vaccine availability, accessibility of vaccination services, cost, distance to vaccination points, timing of vaccination campaigns, lack of regular veterinary services, competing livestock-management priorities, previous experience, or other contextual barriers [20].

The proportion of participants who had heard of anthrax (93.7%) was similar to that reported in Kazo, Uganda (94%) [24], and somewhat lower than the 99% reported in a Kenyan study [25]. Other studies reported lower awareness, including 81% in Zambia [20] and 88% in another setting [27]. Such differences may reflect variation in local disease experience, information sources, study populations and the wording of survey questions; direct comparisons should therefore be made cautiously. In the present study, veterinary professionals were the most frequently reported information source, supporting the potential value of veterinary services as a channel for community education. However, the low proportion who identified the causative agent indicates that awareness messages may need to explain not only the name of the disease but also its cause and routes of transmission. Similar gaps in knowledge of the aetiology of anthrax have been reported in northern Ethiopia and Zambia [18,20].

Participants demonstrated varying levels of knowledge about transmission and prevention. Although many recognised that anthrax can pass from animals to humans, fewer identified specific transmission routes or human clinical signs. The reported knowledge of prevention in animals was higher than knowledge of prevention in humans, which may reflect participants’ closer day-to-day contact with livestock and the greater visibility of animal-health interventions. This interpretation is plausible but cannot be established from the cross-sectional data, and the study did not demonstrate that occupation or previous direct exposure caused these differences. The finding that some respondents identified inappropriate or incomplete preventive measures also indicates that education should be specific and practical, covering safe handling of sick or dead animals, avoidance of meat from animals that die unexpectedly, prompt notification of veterinary authorities and appropriate carcass management.

Most respondents considered anthrax serious in animals, whereas a smaller proportion considered it serious in humans and some reported that they did not know. Perceived seriousness is subjective and may depend on respondents’ experience of animal losses, awareness of human cases, access to reliable information and understanding of zoonotic transmission. The questionnaire’s general wording does not establish what respondents meant by ‘serious’; these responses should therefore be interpreted as perceptions rather than objective assessments of disease severity. Likewise, reports of sudden death as a sign of anthrax should not be interpreted as evidence that anthrax caused a corresponding proportion of animal deaths, because sudden death is not specific to anthrax and the survey did not confirm the cases respondents had observed.

Vaccination was widely recognised as an important preventive measure, and many respondents reported that their animals had been vaccinated. However, reported knowledge and perceived importance did not consistently translate into vaccination at the recommended time or frequency. Participants identified lack of information about vaccination campaigns and distance from vaccination centres as barriers, while more than half of the responses were classified as ‘other’. The discrepancy between recognising the benefits of vaccination and reporting barriers to uptake highlights that information alone may be insufficient when access, timing, cost, livestock-management demands or other local circumstances constrain behaviour. Differences between reports from Zambia and Uganda, where vaccination practices have varied [24,26], may also reflect differences in service delivery and study context rather than knowledge alone.

Carcass management is another important area for prevention. A proportion of participants reported burying or burning carcasses or notifying veterinary authorities when an animal died suddenly, but some reported sharing or consuming the meat or disposing of carcasses in the environment. These reported practices are concerning because handling or consuming products from an infected animal can expose people to *B. anthracis*, while inappropriate carcass disposal may contaminate the environment. Human anthrax cases associated with animal contact or consumption of contaminated meat have been documented in Ethiopia [12,13,17,30,31]. Nonetheless, the present survey relied on respondents’ reports and did not verify the cause of the animals’ deaths; these findings should be interpreted as descriptions of reported responses to sudden animal death, not as confirmation that the animals had anthrax. Community outreach should emphasise avoiding slaughter or consumption of animals found dead or suspected of anthrax, limiting contact with carcasses, and promptly notifying veterinary authorities so that appropriate investigation and disposal can be arranged.

Knowledge scores differed across some demographic groups, with statistically significant differences reported by gender, education and occupation. These associations may indicate that educational opportunities, work-related exposure and access to information are relevant to anthrax knowledge, but the cross-sectional design cannot establish causal relationships. The sample was predominantly male and included a high proportion of farmers. This distribution may reflect recruitment in livestock-owning communities and local divisions of labour, but women also participate in animal husbandry in many settings. The low number of female participants may therefore reflect recruitment or participation patterns as well as broader social roles, and it limits the precision of comparisons by sex. Because the sampling was purposive rather than random, differences between the sample and the wider population may also have influenced the observed associations. These results should not be interpreted as showing that women are inherently less knowledgeable or less engaged in animal health.

Using the study’s operational cut-offs, 60.2% of participants were classified as having adequate knowledge and approximately half were classified as having positive attitudes or good practices. These proportions are broadly comparable with some previous KAP studies, including reports from Bangladesh, northern Ethiopia and Nigeria [18,21,32], but comparisons are limited by potential differences in questionnaire content, scoring rules, participant selection and local circumstances. In addition, the cut-offs used here are not established diagnostic thresholds. The continuous scores and item-level responses should therefore be considered alongside the dichotomised categories.

The positive correlations between knowledge and attitude (r = 0.690), knowledge and practice (r = 0.417), and attitude and practice (r = 0.378) indicate that higher scores in one domain tended to accompany higher scores in the others. These correlations do not establish that knowledge causes safer practices, and the moderate associations between knowledge/practice and attitude/practice suggest that other factors may also influence reported behaviour. Practical barriers, access to veterinary services, economic pressures, social norms and previous experience may be relevant, but the present study cannot determine their individual effects unless these factors were directly measured. The findings support a need for One Health approach across human and animal health sectors that combines clear community communication with accessible veterinary services and locally appropriate guidance on vaccination, safe carcass handling and reporting of suspected cases.

## Conclusion

In this study, knowledge, attitude, and practices (KAP) towards anthrax among communities were addressed. Most participants understood that anthrax can be transmitted from animals to humans, and a few respondents correctly identified germs as the causative agent of anthrax. The majority of the respondents had adequate knowledge and a positive attitude towards anthrax among communities in southern and southwestern Ethiopia. There was a practical gap in terms of carcass management when animals die suddenly, and there is a need for community training and sensitization. In general, our findings provided essential insights into community awareness and preventive practices, which are critical for strengthening anthrax control and prevention strategies.

### Limitation of the study

This study has several limitations. First, the use of purposive, non-probability sampling and the selection of districts and kebeles partly on the basis of previous outbreaks and logistical feasibility may have introduced selection bias. The participants may differ from residents of other communities, including those in areas with different levels of anthrax exposure or access to veterinary services; the results are therefore not statistically generalisable to all communities in Ethiopia. Second, the sample included relatively few women. Although this may partly reflect the recruitment setting and local divisions of livestock-related work, women’s involvement in animal husbandry means that their perspectives may be underrepresented.

Third, the questionnaire was adapted from previous work, translated and pretested, but formal reliability and validity assessments are not documented in the available study materials. Face-to-face interviewing may have introduced interviewer or social-desirability effects, and responses about prior human or animal anthrax cases were not independently verified. Fourth, the study relied on self-reported knowledge and practices, which may be affected by recall and interpretation of the questions. In particular, respondents’ reports of sudden animal death do not confirm anthrax infection, and the perceived seriousness of anthrax is a subjective measure.

Fifth, the KAP categories depend on operational cut-offs that may affect the proportion classified as having adequate knowledge, positive attitudes or good practices. The attitude scoring criteria require verification because the stated score threshold and percentage threshold are inconsistent. In addition, the available reporting contains discrepancies in some demographic and livestock-management figures and in the denominator for a conditional question; these must be checked against the original dataset before publication.

Finally, the large ‘other’ category for barriers to vaccination could not be interpreted from the information currently reported. These limitations should be resolved or explicitly acknowledged in the final version.

## Supporting information

S1 Table Questionnaires. (DOCX)
