# Supplementary material for: Knowledge, attitude, and practices towards anthrax among Communities in the Southern and South-western Ethiopia
Source: PLoS Negl Trop Dis. 2026 Oct 30;20(10):e0014848. doi: 10.1371/journal.pntd.0014848 (PMC13739230; doi:10.1371/journal.pntd.0014848)
Supplement: Supplementary file 1 [file pntd.0014848.s001.docx]

**S 1 Table**: Distribution of knowledge, attitude and practice mean scores among respondents toward anthrax in one-way ANOVA.

| Demographic | Categories | Knowledge | | Attitude | | Practice | |
| --- | --- | --- | --- | --- | --- | --- | --- |
|  |  | Mean±SD* score | P-value | Mean±SD^*^ score | P-value | Mean±SD^*^ score | P-value |
| Age (years) | 19-32 | 5.80±2.58 | 0.692 | 5.33±1.2 | 0.796 | 2.28±1.21 | 0.831 |
|  | 33-47 | 6.11±2.65 |  | 5.39±1.2 |  | 2.41±1.26 |  |
|  | 48-62 | 5.77±2.67 |  | 5.50±1.28 |  | 2.39±1.25 |  |
|  | 63-77 | 5.65±2.39 |  | 5.43±1.16 |  | 2.30±1.36 |  |
| Gender: | Female | 4.18±3.13 | <0.001 | 4.68±1.66 | p<0.000 | 1.93±1.59 | 0.061 |
|  | Male | 6.04±2.52 |  | 5.45±1.15 |  | 2.39±1.21 |  |
| Level of education | Diploma | 7.28±2.00 | p<0.001 | 5.45±1.15 | p<0.001 | 2.56±0.91 | p<0.000 |
|  | Degree | 8.11±1.76 |  | 5.69±0.80 |  | 3.00±0.71 |  |
|  | Junior: 5-8 | 5.81±2.70 |  | 6.33±0.71 |  | 2.31±1.22 |  |
|  | No formal education | 5.07±2.64 |  | 5.23±1.25 |  | 1.91±1.31 |  |
|  | Primary: 1-4 | 5.39±2.83 |  | 5.26±1.31 |  | 2.37±1.23 |  |
|  | Secondary school: 9-12 | 6.60±2.03 |  | 5.29±1.43 |  | 2.83±1.19 |  |
| Occupation | Merchandiser | 6.43±2.47 | <0.001 | 5.50±1.02 | 0.105 | 2.71±1.27 | 0.227 |
|  | Church leader | 6.38±2.00 |  | 5.50±0.73 |  | 2.63±1.02 |  |
|  | Daily worker | 4.67±2.08 |  | 4.67±1.15 |  | 1.67±0.58 |  |
|  | Farmer | 5.61±2.66 |  | 5.33±1.27 |  | 2.28±1.29 |  |
|  | Government employed | 7.89±1.58 |  | 5.92±0.95 |  | 2.68±0.97 |  |
|  | Student | 6.29±2.14 |  | _5.43±0.53_ |  | 2.71±0.95 |  |
|  | Total | 5.90±2.61 |  | _5.40±1.21_ |  | 2.35±1.25 |  |

- Standard deviation
