# Supplementary material for: Knowledge, attitude, and practices towards anthrax among Communities in the Southern and South-western Ethiopia
Source: PLoS Negl Trop Dis. 2026 Oct 30;20(10):e0014848. doi: 10.1371/journal.pntd.0014848 (PMC13739230; doi:10.1371/journal.pntd.0014848)
Supplement: Supplementary file 2 [file pntd.0014848.s002.docx]

**Cover Letter**

Dear Editor,

We appreciate you and the reviewers for your precious time in reviewing our paper and providing valuable comments. It was your valuable and insightful comments that led to possible improvements in the current version. We have carefully considered the comments and tried our best to address every one of them. We hope the manuscript after careful revisions meet your high standards. We welcome further constructive comments if any.

Below we provide the point-by-point responses.

**Sincerely,**

**PLOS Global Public Health Reviewer Comment 1**

For example, it is not clear why you took a number of continuous measures (i.e., knowledge, attitudes, and practices scores) and dichotomised them in addition to treating them as continuous. You refer to “Bloom’s cutoffs”, but this term refers to criteria used to identify mastery in students after a course of study in a given discipline and splitting these scores into arbitrary categories is not appropriate.

**Response 1**

We thank the reviewer for this important comment and for highlighting the conceptual issues related to dichotomising continuous KAP scores.

In the original version of the manuscript, knowledge, attitude, and practice (KAP) scores were analyzed both as continuous variables and as dichotomous outcomes using Bloom’s cut-off points, following approaches commonly applied in KAP studies to facilitate interpretation and comparison across studies. However, we acknowledge the reviewer’s concern that Bloom’s taxonomy was originally developed for educational assessment and mastery learning, and that its direct application to KAP survey data may lack a strong theoretical basis without a formal validation of mastery thresholds.

In response to this comment, we have revised the manuscript to prioritize the analysis and interpretation of KAP scores as continuous variables. The dichotomization of KAP scores has been removed, and all references to Bloom’s cut-offs have been deleted from the Methods, Results, and Discussion sections. This change avoids arbitrary categorization and preserves the full variability and information contained in the data.

Where categorical summaries are presented for descriptive purposes, this is now clearly stated, and no inferential conclusions are drawn from dichotomized outcomes.

**PLOS Global Public Health Reviewer Comment 2**

Chi square is suitable for data where there are distinct, categorical outcomes, but not where the dependent variable is continuous.

**Response 2**

We agree with the reviewer and appreciate this clarification.

Following the revision described above, chi-square tests are no longer used for analyses involving continuous KAP scores. Instead, appropriate statistical methods (One-way variance analysis (ANOVA) for continuous outcomes have been applied as specified in the revised Methods section). The Statistical analysis section has been revised accordingly to clearly describe the updated analytical approach and to ensure alignment between the nature of the dependent variables and the statistical tests employed.

**One Health Journal Editor comments(if applicable):**

Although your manuscript falls within the aim and scope of this journal, it is being declined due to lack of sufficient novelty. We receive a much larger number of papers than we are able to accept.
