# Supplementary material for: Knowledge, attitude, and practices towards anthrax among Communities in the Southern and South-western Ethiopia
Source: PLoS Negl Trop Dis. 2026 Oct 30;20(10):e0014848. doi: 10.1371/journal.pntd.0014848 (PMC13739230; doi:10.1371/journal.pntd.0014848)
Supplement: Supplementary file 3 [file pntd.0014848.s003.docx]

**Manuscript: Knowledge, attitudes, and practices regarding anthrax in selected areas of southern and southwestern Ethiopia**

Dear Editor,

We thank the Editor and the reviewers for their careful assessment of our manuscript and for the constructive comments. We have revised the manuscript to improve the description of the study methods, clarify the interpretation of the findings, and acknowledge the limitations of the sampling strategy and KAP scoring approach. Below, we respond point by point.

# A. Response to the Editor

## Editorial comments 1–4: author contributions, consent, Supporting Information, and funding disclosures

Response: We thank the Editor for these requests. We have reviewed the CRediT (Contributor Roles Taxonomy) author contributions for all co-authors and updated the relevant information in the “Add/Edit/Remove Authors” section of the submission system. We have ensured that each author's contributions are accurately and fully acknowledged according to their actual involvement in the study.

Oral consent was obtained from all respondents before data collection was carried out and stated in the manuscript under Ethical consideration subheading highlighted with yellow and the first question asked for their consent to participants in this study is identified as Supporting Information File S1 Table.

We have revised the relevant figure captions to include the corresponding attribution, to ensure that the source or attribution is clearly acknowledged in the manuscript. In addition, the legend/description for the Supporting Information has been added after the references, and the questionnaire is identified as Supporting Information File S1 Table. The Financial Disclosure statement has also been revised to clarify the role of the funders.

# B. Reviewer #1

## Comment 1. Novelty and knowledge gap

Response: We agree that the contribution of the study should be distinguished more clearly from previous KAP surveys. We have revised the Introduction to explain that, although anthrax KAP studies have been conducted in other settings, local evidence remains important because knowledge and preventive practices are shaped by local livestock-management practices, access to veterinary services, previous exposure to outbreaks, and sociocultural and economic circumstances. The present study provides a description of KAP patterns among respondents recruited from selected districts in southern and southwestern Ethiopia and identifies areas in which reported awareness does not necessarily correspond to specific knowledge or preventive practices.

We have also clarified that the findings are intended to inform context-specific risk communication and prevention planning, rather than to establish nationally representative estimates. The contribution of the study should therefore be interpreted considering its purposive, non-probability sampling design.

## Comment 2. Sampling strategy, representativeness, and selection bias

Response: We have clarified in the Methods that a community-based cross-sectional survey was conducted from February to November 2025 using face-to-face interviews and a non-probability, purposive sampling approach. Study areas were selected in relation to the history of anthrax outbreaks, and participants were recruited from the selected study communities based on the study’s eligibility criteria, availability, and willingness to participate.

We acknowledge that this sampling approach does not provide each member of the target population with a known probability of selection and therefore cannot establish that the sample is representative of the wider population. The high proportion of male respondents and farmers may reflect the recruitment strategy and the study’s focus on communities with livestock-related exposure; however, the available data do not allow us to determine the relative contribution of local gender roles, occupational patterns, or selection effects. We have added this issue to the Limitations and have avoided generalising the estimates beyond the surveyed participants and study areas.

## Comment 3. Discussion: comparison, sociocultural interpretation, and public-health implications

Response: We have revised the Discussion to move beyond a repetition of the Results. The revised text compares the observed awareness and knowledge findings with studies cited in the manuscript from Ethiopia and other countries, while noting that differences in study populations, questionnaires, scoring systems, and sampling methods limit direct comparisons. We have also discussed why awareness of anthrax may coexist with gaps in knowledge about its cause, transmission, clinical manifestations, and prevention.

The Discussion now treats reported practices as self-reported behaviours and distinguishes respondents’ recognition of a possible clinical sign from evidence that anthrax caused a particular animal death. We have also discussed the potential gap between knowledge and vaccination practices without attributing that gap to specific barriers unless those barriers were reported in the data. The public-health implications are framed as priorities for locally adapted communication, veterinary engagement, safe carcass handling, and accessible prevention services, rather than as causal conclusions from this cross-sectional survey.

# C. Reviewer #2 — questionnaire and methods

## Comment 1. Reliability and validity of the questionnaire

Response: We agree that the questionnaire’s development and pretesting should be described clearly. The questionnaire was informed by relevant literature and previously published anthrax KAP questionnaires, adapted to the Ethiopian context, translated into Amharic and Afaan Oromo, and pilot-tested in a rural community in Sebeta among individuals who were not included in the final study. The pilot test was used to assess clarity, comprehensibility, relevance, wording, sequence, and the time required to complete the questionnaire; wording and sequence were adjusted in response to feedback.

The available study documentation does not report formal psychometric validation or a quantitative reliability assessment. We therefore do not claim that such testing was performed. This limitation is acknowledged, and the pilot test is described as a pretest of comprehensibility and feasibility rather than proof of reliability or construct validity. Data cross-checking is described separately as a data-quality procedure, not as a measure of questionnaire reliability.

## Comment 2. Appropriateness for participants with different educational backgrounds

Response: The questionnaire was adapted for the target population and translated into Amharic and Afaan Oromo. For participants who did not speak either language, the authors report that a translator was used. The questionnaire used simple wording and commonly understood concepts relevant to anthrax recognition, transmission, risk factors, prevention, carcass handling, and actions following suspected cases. Trained data collectors administered the questions and provided clarification where necessary.

## Comment 3. Face-to-face interviews and potential bias

Response: Face-to-face interviews were used because the study population included people with different levels of formal education and literacy, and a self-administered questionnaire could have excluded some eligible participants or increased misunderstanding of questions. A structured questionnaire was used to maintain consistency across interviews.

## Comment 4. Choice of p in the sample-size calculation and distinction between awareness and adequate knowledge

Response: We agree that awareness of anthrax and adequate knowledge of anthrax are distinct outcomes. In the previous study used to inform the calculation, awareness referred to having heard of or being aware of anthrax; it did not necessarily demonstrate correct or comprehensive knowledge of transmission, clinical manifestations, prevention, or treatment. The 62% estimate was used as an available prior estimate for the sample-size calculation, not as the definition of adequate knowledge in the present study.

## Comment 5. Rationale and limitations of KAP cut-offs

Response: We have expanded the explanation of the KAP cut-offs and cited the prior studies identified by the authors, including Cadmus et al. (2024), where relevant. We acknowledge that the thresholds are operational classifications and are not universally validated measures of adequate knowledge, positive attitudes, or good practices. Dichotomising scores may also obscure differences between participants and can limit comparisons across studies that use different items or scoring systems.

# D. Reviewer #2 — Results and data presentation

## Comment 1. Participant selection and representativeness

Response: Participants were selected purposively, rather than randomly, from the selected study areas. The revised Methods describes the sampling approach and the rationale for selecting areas with a history of anthrax outbreaks. We acknowledge that the resulting sample may differ from the general population in occupation, education, sex, and previous exposure to livestock or anthrax. The sample should not be interpreted as representative of all residents of the surveyed districts or of Ethiopia as a whole. This limitation is now stated in the Discussion/Limitations.

## Comment 2. Percentages for “Who can be infected by anthrax?”

Response: We agree that the presentation could be confusing because the question allowed respondents to identify more than one response category. The percentages should be interpreted as the proportion of respondents selecting each option; they are not mutually exclusive and therefore need not sum to 100%. We have clarified the multiple-response nature of the item in the table note and/or Results text.

## Comment 3. Respondents who had seen or known a person with anthrax

Response: We agree that the original wording could imply that respondents were expected to diagnose anthrax themselves. The question was intended to ask whether respondents had seen or known a person reported or confirmed to have anthrax. The wording has been revised accordingly.

This remains a self-reported measure and does not independently verify the diagnosis of the person concerned. Where the respondent’s source of information or diagnostic confirmation was not recorded, that uncertainty cannot be resolved retrospectively and should be acknowledged when interpreting the finding.

## Comment 4. Sudden death, “Other” clinical signs, and perceived disease severity

Response: The percentage identifying sudden death (46.6%) represents the proportion of respondents who selected it as a possible clinical manifestation of anthrax. It is not an estimate of the proportion of animal deaths caused by anthrax, and we have clarified this distinction in the interpretation.

The “Other” response option was intended to capture signs not listed among the predefined options. The authors’ response indicates that an open-text response such as splenomegaly was included in this category when provided. The corresponding table and text should make clear which additional signs were actually recorded and how they were coded.

We also agree that the question asking whether anthrax is a “serious disease” measures perceived severity, not an objective measure of disease severity. Perceptions may vary with personal experience, occupation, prior exposure, economic losses, and perceived consequences for people and animals. The finding is therefore interpreted as an attitude/perception measure rather than an epidemiological assessment of severity.

## Comment 5. Awareness, perceived severity, vaccination, and the “Other” vaccination-barrier category

Response: The authors’ data indicate that 24 participants had never heard of anthrax and that 45 reported not knowing how serious anthrax was for humans. The response letter states that the 24 participants were also among those who did not know its seriousness. This overlap should be reported only if confirmed from the respondent-level data; the revised Results should state the exact relationship clearly.

Regarding reasons for not consistently vaccinating animals, the “Other” category accounted for 55.8% of responses. The authors’ response mentions additional entries such as “causes death during drought season,” “swelling of the neck during farming time,” and “causes abortion during pregnancy.” These appear to describe perceived effects or concerns and should not be treated as verified reasons for non-vaccination unless the questionnaire responses establish that interpretation. The table and text should identify what participants actually reported and distinguish verbatim or coded responses from author interpretation.

We agree that knowledge of anthrax and vaccination does not necessarily translate into consistent vaccination behaviour. Access to services, timing, cost, distance, vaccine availability, and competing livestock-management priorities are plausible explanations, but they should be presented as possible contextual factors rather than study findings unless they were directly measured. The Discussion has been revised to make this distinction.

## Comment 6. Relevance and placement of the grazing-system variable

Response: We agree that grazing system is primarily a livestock-management characteristic and not necessarily a direct measure of preventive practice. It was collected to provide context about livestock management and potential environmental exposure. We have clarified its intended role and recommend presenting it with participant/livestock-management characteristics rather than counting it as a practice item, unless the original study protocol explicitly defined it as part of the practice domain.

## Comment 7. Meaning of “level of disease outbreak”

Response: The item was intended to capture respondents’ perception or reported understanding of the extent of an anthrax outbreak in their community, rather than their ability to apply a formal epidemiological outbreak definition. We agree that the phrase may be interpreted differently according to education, occupation, prior exposure, and experience with livestock or animal-health services.

The questionnaire wording and Methods description have been clarified to the extent supported by the study documentation. Because a standardised explanation given during interviews cannot be verified from the questionnaire alone, the final manuscript should describe the wording and interviewer instructions accurately and acknowledge potential variation in interpretation.

## Comment 8. Comparing KAP scores and subgroup analyses

Response: We agree that raw scores from domains containing different numbers of items should not be interpreted as equivalent scales. The manuscript reports knowledge, attitude, and practice scores on their respective item ranges, and the Discussion now cautions against direct comparison of their magnitudes. The associations between demographic characteristics and KAP scores should be interpreted within each domain.

## Comment 9. Availability of the questionnaire and skip patterns

Response: The questionnaire has been included as Supporting Information File’’ S1 Table ‘’ so readers can examine the wording, response options, and skip patterns. The Methods now states that skip patterns were considered in scoring. Questions that were not applicable because of a preceding response should not automatically be treated as incorrect.

# E. Reviewer #2 — Discussion of participant characteristics and differences across items

## Comment. Sex, occupation, sociocultural context, and possible selection bias

Response: We have revised the Discussion to reiterate that the sample was predominantly male (91.0%) and that farmers accounted for 78.4% of respondents, while 27.8% (n = 108) had no formal education, according to the values reported in the manuscript. These characteristics are relevant to interpreting the KAP findings, but the study design does not establish why the sample had this distribution.

We acknowledge that women may also participate actively in livestock care and that recruitment procedures may have underrepresented some groups. The revised Discussion therefore treats the observed sex and occupational distribution as a characteristic of the recruited sample and a potential source of selection bias, rather than assuming that it reflects the whole local population. We have also noted that occupation, education, and previous exposure may influence responses to individual questions, although the present data do not establish causal explanations for all item-level differences.

# F. Reviewer #3

## Comment 1. Whether the study requires a hypothesis

Response: We thank the reviewer for this observation and agree that the study is primarily a descriptive, community-based cross-sectional survey of knowledge, attitudes, and practices regarding anthrax in selected areas of Ethiopia. We have retained the clearly stated study objectives and have avoided presenting the study as a hypothesis-testing investigation beyond the analyses actually performed.

## Comment 2. Multiple-response questions and frequency calculations

Response: We agree that the handling of questions permitting more than one response must be explicit. For such items, each selected option is counted as a response for that category; consequently, one participant may contribute to more than one category, and percentages across categories may exceed 100%. The revised table notes and Results text clarify this point.

## Comment 3. English-language errors and typos

Response: We thank the reviewer for noting the language issues. The manuscript has been edited for grammar, clarity, terminology, and consistency. We will conduct a final language and table-proofreading pass after all numerical and methodological corrections have been completed.

## Comment 4. Public-health relevance

Response: We thank the reviewer for the positive assessment of the public-health relevance of the study. The revised Discussion retains this focus while qualifying the implications in view of the purposive sampling design and the self-reported nature of the data.
