# Supplementary material for: Knowledge, attitude, and practices towards anthrax among Communities in the Southern and South-western Ethiopia
Source: PLoS Negl Trop Dis. 2026 Oct 30;20(10):e0014848. doi: 10.1371/journal.pntd.0014848 (PMC13739230; doi:10.1371/journal.pntd.0014848)
Supplement: Peer review history

# Author comment: Author response to Decision Letter 0

**DOI:** 10.1371/journal.pntd.0014848.r001

4 Mar 2026

Attachment Submitted filename: Response file.docx

# Peer review: Decision Letter 0

**DOI:** 10.1371/journal.pntd.0014848.r002

**Authors:** Richard Bowen, Ana LTO Nascimento

5 Oct 2026

PNTD-D-26-00445Knowledge, Attitude, and Practices towards Anthrax among Communities in the Southern and South-western Ethiopia.PLOS Neglected Tropical DiseasesDear Dr. Bulto,Thank you for submitting your manuscript to PLOS Neglected Tropical Diseases. After careful consideration, we feel that it has merit but does not fully meet PLOS Neglected Tropical Diseases's publication criteria as it currently stands. Therefore, we invite you to submit a revised version of the manuscript that addresses the points raised during the review process.Please submit your revised manuscript in 10 days. If you will need more time than this to complete your revisions, please reply to this message or contact the journal office at plosntds@plos.org. When you're ready to submit your revision, log on to https://www.editorialmanager.com/pntd/ and select the 'Submissions Needing Revision' folder to locate your manuscript file.Please include the following items when submitting your revised manuscript:* A letter that responds to each point raised by the editor and reviewer(s). You should upload this letter as a separate file labeled 'Response to Reviewers'. This file does not need to include responses to any formatting updates and technical items listed in the 'Journal Requirements' section below.* A marked-up copy of your manuscript that highlights changes made to the original version. You should upload this as a separate file labeled 'Revised Manuscript with Track Changes'.* An unmarked version of your revised paper without tracked changes. You should upload this as a separate file labeled 'Manuscript'.If you would like to make changes to your financial disclosure, competing interests statement, or data availability statement, please make these updates within the submission form at the time of resubmission. Guidelines for resubmitting your figure files are available below the reviewer comments at the end of this letter.

As the corresponding author, your ORCID iD is verified in the submission system and will appear in the published article. PLOS supports the use of ORCID, and we encourage all coauthors to register for an ORCID iD and use it as well. Please encourage your coauthors to verify their ORCID iD within the submission system before final acceptance, as unverified ORCID iDs will not appear in the published article. Only the individual author can complete the verification step; PLOS staff cannot verify ORCID iDs on behalf of authors.We look forward to receiving your revised manuscript.Kind regards,Richard A. Bowen, DVM PhDAcademic EditorPLOS Neglected Tropical DiseasesAna LTO NascimentoSection EditorPLOS Neglected Tropical Diseases

Kamhawi

co-Editor-in-Chief

PLOS Neglected Tropical Diseases

orcid.org/0000-0003-4304-636XX

Paul Brindley

co-Editor-in-Chief

PLOS Neglected Tropical Diseases

orcid.org/0000-0003-1765-0002

**Additional Editor Comments (if provided):** Your manuscript has now been reviewed by 3 experts, each of whom believe it is a valuable study and worthy of publication. Each reviewer had some minor suggestions to improve clarity and quality - please evaluate these comments, edit your manuscript to address these comments, then resubmit along with a point-by-point response to the reviewer comments. Thank you!**Journal Requirements:**

If the reviewer comments include a recommendation to cite specific previously published works, please review and evaluate these publications to determine whether they are relevant and should be cited. There is no requirement to cite these works unless the editor has indicated otherwise.

1) Please ensure that the CRediT author contributions listed for every co-author are completed accurately and in full.

At this stage, the following Authors/Authors require contributions: Abebe Olani, Domenico Galante, Matios Lakew, Tigist Ashagre, Taferi Benti, Gizat Almaw, Fasil Aklilu, Getnet Abie.Mekonnen, Tesfaye Rufael, Nigatu Kebede, Aklilu Feleke Haile, and Tadesse Eguale. Please ensure that the full contributions of each author are acknowledged in the "Add/Edit/Remove Authors" section of our submission form.

The list of CRediT author contributions may be found here: https://journals.plos.org/plosntds/s/authorship#loc-author-contributions

2) We have noticed that you have uploaded Supporting Information files, but you have not included a list of legends. Please add a full list of legends for your Supporting Information files after the references list.

3) Please amend your detailed Financial Disclosure statement. This is published with the article. It must therefore be completed in full sentences and contain the exact wording you wish to be published.

i) State what role the funders took in the study. If the funders had no role in your study, please state: "The funders had no role in study design, data collection and analysis, decision to publish, or preparation of the manuscript."

ii) If any authors received a salary from any of your funders, please state which authors and which funders..

If you did not receive any funding for this study, please simply state: u201cThe authors received no specific funding for this work.u201d

4) We have amended your Competing Interest statement to comply with journal style. We kindly ask that you double check the statement and let us know if anything is incorrect.

**Reviewers' comments:** Reviewer's Responses to Questions

**Key Review Criteria Required for Acceptance?**

As you describe the new analyses required for acceptance, please consider the following:

**Methods**

-Are the objectives of the study clearly articulated with a clear testable hypothesis stated?

-Is the study design appropriate to address the stated objectives?

-Is the population clearly described and appropriate for the hypothesis being tested?

-Is the sample size sufficient to ensure adequate power to address the hypothesis being tested?

-Were correct statistical analysis used to support conclusions?

-Are there concerns about ethical or regulatory requirements being met?

Reviewer #1: (No Response)

Reviewer #2: These comments concern a Knowledge–Attitude–Practice (KAP) study. Therefore, the authors are requested to address the following questions:

1. Reliability and validity of the questionnaire: The authors are requested to clarify the reliability of the questionnaire used in this study. Has this questionnaire previously been administered to the same or a similar target population, and if so, what was its level of reliability? Alternatively, was the questionnaire pilot-tested in a sample of the target population? Has its reliability been assessed by the researchers among a population similar to the population targeted in the present study?

2. Appropriateness of the questionnaire for the study population: Is the questionnaire appropriate for the participants who were interviewed? Knowledge, skills, and attitudes may depend on the participants’ educational level, as well as the relevance of the information being assessed to the target population. Therefore, the authors should clarify how the questionnaire was developed and whether its content and wording were appropriate for the characteristics and level of understanding of the study participants.

3. Face-to-face interviews versus self-administered checklists: Why was a face-to-face interview used rather than a questionnaire or checklist that could be completed independently by the participants? Could the use of face-to-face interviews have introduced bias and affected the accuracy of the study findings?

4. Selection of p for the sample size calculation: In the sample size calculation, p represents the expected proportion of the outcome of interest, for example, the proportion of participants who demonstrate correct practice among those with adequate knowledge. Why, then, did the authors select p based on the proportion of community respondents who were aware of anthrax in a previous study conducted in northern Ethiopia? If this previous study was used as the basis for selecting p, the authors should clarify how “being aware of anthrax” was defined. Did “being aware” simply mean having heard of anthrax, or did it indicate that the respondent had correct or adequate knowledge about anthrax? In particular, “awareness of anthrax” should be distinguished from “adequate knowledge of anthrax,” as these are not necessarily equivalent concepts. A participant may have heard of anthrax without having sufficient or accurate knowledge about its transmission, clinical manifestations, prevention, or treatment.

5. In the section “Scoring methods of knowledge, attitude and practices”: the authors are requested to provide the rationale for selecting the cut-off point, including information on its reliability and the supporting references, such as Cadmus et al. (2024). Alternatively, the authors may provide a discussion of the rationale and limitations of this cut-off selection in the Discussion section.

Reviewer #3: The authors have clearly stated the study objectives. However, I do not think the nature of this study necessarily warrants a hypothesis; the study is simply a survey of knowledge, attitudes, and practices regarding anthrax in certain areas of Ethiopia. The authors have clearly described the background of the study subjects, and the sample size, which the authors determined by a statistical method. The statistical analyses appear sufficient for the study. The authors state that they complied with the rules and regulations that govern ethical standards, such as those established by the relevant institutional review board.

**********

**Results**

-Does the analysis presented match the analysis plan?

-Are the results clearly and completely presented?

-Are the figures (Tables, Images) of sufficient quality for clarity?

Reviewer #1: (No Response)

Reviewer #2: 1. The study was conducted among a population with diverse occupations and educational backgrounds. Both occupation and educational level can have a substantial influence on KAP. Therefore, could the authors provide further clarification on how the interview participants were selected? Were the participants selected randomly or purposively? If random sampling was used, did the characteristics of the study participants, particularly in terms of occupation and educational level, correspond to the distribution of these characteristics in the general population of the areas surveyed?

2. For the question “Who can be infected by anthrax?”, there may be some confusion regarding the percentages, as their sum exceeds 100%.

3. Additionally, the question “Have you ever seen a person infected with anthrax?” is unclear. How could respondents be certain that the person they had seen was actually infected with anthrax?

4. One of the response options for the question “Clinical signs/symptoms of an animal infected with anthrax” was “Sudden death,” which accounted for as much as 46.6% of responses. Does this suggest that anthrax is one of the major causes of animal mortality in the surveyed area? Furthermore, what does the response option “Other” refer to? Does it indicate other clinical signs or symptoms that were not included among the listed response options?

Each individual may have a different perception of what constitutes a “serious disease.” Therefore, is the question regarding whether anthrax is a serious disease too general or subjective? The perceived severity of the disease may also depend on the extent of its impact and the losses it causes in both animals and humans.

5. In addition, the study included 24 participants who had never heard of anthrax, yet 45 participants reported that they did not know how serious anthrax was for humans. Were the 24 participants who had never heard of anthrax also included among the 45 participants who did not know about its severity in humans? Vaccination is another important preventive measure. Therefore, in response to the question “If you do not always take your animals for vaccination, what are the reasons?”, the proportion selecting “Other” was as high as 55.8%. Did the authors collect or analyze any additional information regarding these “other” reasons? Such information could be useful for identifying barriers to animal vaccination and improving vaccination coverage in the community. This finding also appears somewhat inconsistent with the high levels of knowledge reported elsewhere in the study: 91.1% of participants knew that anthrax is dangerous to animals, 46.6% identified sudden death as a clinical sign of anthrax in animals, and 81.4% knew that vaccination can help prevent anthrax in animals. The authors are therefore encouraged to discuss this apparent discrepancy and explore possible reasons why relatively high levels of knowledge did not necessarily translate into consistent vaccination practices.

6. In the Practice section, what is the relevance of the question regarding the livestock grazing system to anthrax-related practices? Alternatively, is this question intended to describe the demographic or livestock-management characteristics of the study population? If the latter is the case, it may be more appropriate to present it in the section describing participants’ characteristics rather than in the Practice section, particularly since the authors stated that there were five questions assessing practices.

7. What is the definition of the level of disease outbreak used for the respondents? Is this concept clearly defined and understandable to the participants? Could the respondents’ ability to answer this question be influenced by their occupation or educational level?

8. When assessing the association between knowledge, attitudes, and practices, the numbers of questions in the knowledge, attitude, and practice domains are different. Therefore, is presenting the scores as mean ± SD appropriate and valid for comparing these domains? Would it be more appropriate to present the results as the percentage of correct responses or as standardized scores, given the different numbers of questions in each domain?

In addition, if feasible, the authors could consider using appropriate statistical tests to compare the differences between the two groups, particularly for the smaller subgroups, rather than relying solely on descriptive comparisons.

9. I could not find the interview questionnaire. I noticed that, for some questions, participants were instructed to proceed to the next question only if they answered “Yes” to the preceding question. Could the authors clarify whether this skip pattern or conditional questioning was taken into account in the scoring of the KAP domains?

Files, images, and data analysis are unavailable until usage resets at 5:06 PM. Continue chatting with text only, or upgrade for more access.

Try Plus free

Reviewer #3: Yes to all three statements above.

**********

**Conclusions**

-Are the conclusions supported by the data presented?

-Are the limitations of analysis clearly described?

-Do the authors discuss how these data can be helpful to advance our understanding of the topic under study?

-Is public health relevance addressed?

Reviewer #1: (No Response)

Reviewer #2: In the Discussion section, I suggest that the authors reiterate the characteristics of the study participants, particularly the high proportion of farmers, which may partly explain the predominance of male participants in the study. However, the authors should also discuss the reasons for this distribution in relation to the demographic and sociocultural characteristics of the local population. In livestock farming, women may also be actively involved in animal husbandry and related activities. Therefore, could there have been any selection bias or confounding related to the selection of interview participants, particularly with respect to sex and occupation?

In addition, there appear to be differences in the responses across the individual questions, as described in the Results section. The authors could further explore and discuss possible explanations for these differences in the Discussion. In particular, it would be useful to consider whether these differences may be related to participants’ occupation, educational level, prior exposure to anthrax, or differences in the nature and difficulty of the individual questions.

Reviewer #3: Yes. The authors have effectively addressed the public health relevance of their study.

**********

**Editorial and Data Presentation Modifications?**

Use this section for editorial suggestions as well as relatively minor modifications of existing data that would enhance clarity. If the only modifications needed are minor and/or editorial, you may wish to recommend “Minor Revision” or “Accept”.

Reviewer #1: (No Response)

Reviewer #2: (No Response)

Reviewer #3: The main confusion for this reviewer is how the sorting of responses to various questions by a given individual was done, as the total number (n) still remains the same, i.e.. 382. For example, if one respondent gives two or more answers to a given question, how do the authors count such answers when they determine the frequencies in their tables? Let's say one person's answer to the question "Who can be infected by anthrax" is only animals, but another person's answer is animals and humans. Would the second person's double response count twice, once for animals and once for humans?

There are also some English errors or typos in the manuscript. I have not corrected those thinking the authors will do that.

**********

**Summary and General Comments**

Use this section to provide overall comments, discuss strengths/weaknesses of the study, novelty, significance, general execution and scholarship. You may also include additional comments for the author, including concerns about dual publication, research ethics, or publication ethics. If requesting major revision, please articulate the new experiments that are needed.

Reviewer #1: Overall, the manuscript addresses an important public health topic and presents potentially valuable data regarding knowledge, attitudes, and practices (KAP) related to the investigated disease/condition. The study includes a relatively comprehensive dataset and attempts to identify associated demographic and behavioral factors. The manuscript is generally well organized, and the authors have clearly invested considerable effort in data collection and revision. However, several methodological, analytical, and presentation issues still require further clarification and improvement before the manuscript can be considered for publication. The following comments are intended to help strengthen the scientific rigor, clarity, and overall impact of the work.

1.The novelty of the study remains insufficiently emphasized throughout the manuscript. Although the study presents KAP data, similar surveys have been widely conducted in different settings, and the manuscript does not clearly explain what specifically distinguishes this work from previous studies. The authors are encouraged to explicitly identify the knowledge gap addressed by this study and clarify how the findings provide new scientific or public health insights beyond descriptive reporting

2.The sampling strategy and representativeness of the study population need further discussion. The manuscript should clearly explain how participants were recruited and whether the sample adequately represents the target population. Potential sources of selection bias, especially if convenience sampling or online recruitment was used, should be acknowledged more explicitly in the limitations section.

3.The Discussion section currently focuses heavily on repeating the study findings rather than critically interpreting them. The authors should expand the discussion by comparing their results more comprehensively with previous regional and international studies, exploring possible sociocultural explanations for the observed patterns, and discussing the broader implications of the findings for public health interventions and policy development.

Reviewer #2: (No Response)

Reviewer #3: In this study the authors report a general survey of people's knowledge, attitudes, and practices regarding anthrax in certain regions of Ethiopia. The study is important because Ethiopia, like many other developing nations, heavily relies on domesticated animals of economic significance.

My main questions about the study appear in the Editorial and Data Presentation section.

**********

PLOS authors have the option to publish the peer review history of their article (what does this mean?). If published, this will include your full peer review and any attached files.

If you choose “no”, your identity will remain anonymous but your review may still be made public.

**Do you want your identity to be public for this peer review?**  For information about this choice, including consent withdrawal, please see our Privacy Policy.

Reviewer #1: **Yes:**  Ahmed Hamdy Ghonaim

Reviewer #2: No

Reviewer #3: No

[NOTE: If reviewer comments were submitted as an attachment file, they will be attached to this email and accessible via the submission site. Please log into your account, locate the manuscript record, and check for the action link "View Attachments". If this link does not appear, there are no attachment files.]**Figure resubmission:** While revising your submission, we strongly recommend that you use PLOS’s NAAS tool (https://ngplosjournals.pagemajik.ai/artanalysis) to test your figure files. NAAS can convert your figure files to the TIFF file type and meet basic requirements (such as print size, resolution), or provide you with a report on issues that do not meet our requirements and that NAAS cannot fix.

After uploading your figures to PLOS’s NAAS tool - https://ngplosjournals.pagemajik.ai/artanalysis, NAAS will process the files provided and display the results in the "Uploaded Files" section of the page as the processing is complete. If the uploaded figures meet our requirements (or NAAS is able to fix the files to meet our requirements), the figure will be marked as "fixed" above. If NAAS is unable to fix the files, a red "failed" label will appear above. When NAAS has confirmed that the figure files meet our requirements, please download the file via the download option, and include these NAAS processed figure files when submitting your revised manuscript. **Reproducibility:** To enhance the reproducibility of your results, we recommend that authors of applicable studies deposit laboratory protocols in protocols.io, where a protocol can be assigned its own identifier (DOI) such that it can be cited independently in the future. Additionally, PLOS ONE offers an option to publish peer-reviewed clinical study protocols. Read more information on sharing protocols at https://plos.org/protocols?utm_medium=editorial-email&utm_source=authorletters&utm_campaign=protocols

# Author comment: Author response to Decision Letter 1

**DOI:** 10.1371/journal.pntd.0014848.r003

20 Oct 2026

Attachment Submitted filename: Response to reviewer.docx

# Editor report: Decision Letter 1

**DOI:** 10.1371/journal.pntd.0014848.r004

**Authors:** Richard Bowen, Ana LTO Nascimento

22 Oct 2026

Dear Dr. Bulto,

We are pleased to inform you that your manuscript 'Knowledge, Attitude, and Practices towards Anthrax among Communities in the Southern and South-western Ethiopia.' has been provisionally accepted for publication in PLOS Neglected Tropical Diseases.

Before your manuscript can be formally accepted you will need to complete some formatting changes, which you will receive in a follow up email. A member of our team will be in touch with a set of requests.

Please note that your manuscript will not be scheduled for publication until you have made the required changes, so a swift response is appreciated.

IMPORTANT: The editorial review process is now complete. PLOS will only permit corrections to spelling, formatting or significant scientific errors from this point onwards. Requests for major changes, or any which affect the scientific understanding of your work, will cause delays to the publication date of your manuscript.

Should you, your institution's press office or the journal office choose to press release your paper, you will automatically be opted out of early publication. We ask that you notify us now if you or your institution is planning to press release the article. All press must be co-ordinated with PLOS.

Thank you again for supporting Open Access publishing; we are looking forward to publishing your work in PLOS Neglected Tropical Diseases.

Best regards,

Richard A. Bowen, DVM PhD

Academic Editor

PLOS Neglected Tropical Diseases

Ana LTO Nascimento

Section Editor

PLOS Neglected Tropical Diseases

Shaden Kamhawi

co-Editor-in-Chief

PLOS Neglected Tropical Diseases

orcid.org/0000-0003-4304-636XX

Paul Brindley

co-Editor-in-Chief

PLOS Neglected Tropical Diseases

orcid.org/0000-0003-1765-0002

***********************************************************

Thank you for carefully and comprehensively addressing reviewer comments for your manuscript. I believe this is now acceptable for publication.

# Editor report: Acceptance letter

**DOI:** 10.1371/journal.pntd.0014848.r005

**Authors:** Richard Bowen, Ana LTO Nascimento

Dear Mr Bulto,

We are delighted to inform you that your manuscript, "Knowledge, Attitude, and Practices towards Anthrax among Communities in the Southern and South-western Ethiopia.," has been formally accepted for publication in PLOS Neglected Tropical Diseases.

We have now passed your article onto the PLOS Production Department who will complete the rest of the publication process. All authors will receive a confirmation email upon publication.

The corresponding author will soon be receiving a typeset proof for review, to ensure errors have not been introduced during production. Please review the PDF proof of your manuscript carefully, as this is the last chance to correct any scientific or type-setting errors. Please note that major changes, or those which affect the scientific understanding of the work, will likely cause delays to the publication date of your manuscript. Note: Proofs for Front Matter articles (Editorial, Viewpoint, Symposium, Review, etc...) are generated on a different schedule and may not be made available as quickly.

Soon after your final files are uploaded, the early version of your manuscript will be published online unless you opted out of this process. The date of the early version will be your article's publication date. The final article will be published to the same URL, and all versions of the paper will be accessible to readers.

For Research Articles, you will receive an invoice from PLOS for your publication fee after your manuscript has reached the completed accept phase. If you receive an email requesting payment before acceptance or for any other service, this may be a phishing scheme. Learn how to identify phishing emails and protect your accounts at https://explore.plos.org/phishing.

Thank you again for supporting open-access publishing; we are looking forward to publishing your work in PLOS Neglected Tropical Diseases.

Best regards,

Shaden Kamhawi

co-Editor-in-Chief

PLOS Neglected Tropical Diseases

Paul Brindley

co-Editor-in-Chief

PLOS Neglected Tropical Diseases
